# Mechanisms of Action of Low-Frequency Pulsed Magnetic Fields in Pain Control

**DOI:** 10.3390/bioengineering13040407

**Published:** 2026-03-31

**Authors:** Marshall Bedder, Alaa Abd-Elsayed

**Affiliations:** 1 Department of Psychiatry and Health Behavior, Wellstar/Medical College of Georgia, Augusta, GA 30912, USA; 2 Department of Anesthesiology, University of Wisconsin, Madison, WI 53706, USA; alaaawny@hotmail.com

**Keywords:** low frequency pulsed magnetic fields, LFPMF, magnetic peripheral nerve stimulation, pain control, neuropathic pain, neuronal blockade, electromagnetic therapy

## Abstract

Low-frequency pulsed magnetic fields (LFPMFs) are a recently developed modality for managing pain and promoting wound healing. The term LFPMF is used to describe low-intensity fields in wound and tissue studies, and is referred to as magnetic peripheral nerve stimulation (mPNS) in pain-related studies. The recent clearance of the first mPNS device for treating pain due to diabetic neuropathy by the FDA marks a watershed event in the clinical acceptance of these modalities. In addition to being within the frequency range of 0.5–100 Hz, the use of electromagnetic fields rather than electrical current, which dissipates in tissues, results in several therapeutic advantages of magnetic fields. These fields permeate tissues and affect a larger area.

## 1. Introduction

Pain control and wound healing are among the most challenging problems in the medical field to treat effectively. For millions of patients worldwide, there is often inadequate relief from the multiple medications used, surgery, and other more invasive treatments that are available. The cost of care is high, and there remains significant interest in alternative medical practices. Electromagnetic approaches have a long history but have only recently been investigated and identified as a possible therapy for these conditions. Low-frequency pulsed magnetic fields (LFPMFs), particularly mPNS, are particularly promising.

Electromagnetic field therapy was utilized in medicine soon after its discovery at the end of the nineteenth century. However, the mechanisms of action have not been well understood until recently. LFPMFs are electromagnetic fields that operate in the range of 0.5 Hz to 100 Hz. This frequency range is what distinguishes these therapies from others in the electromagnetic spectrum. There are several reasons for this, and it has been found that this frequency range promotes biological effects on the body without the negative consequences associated with the higher end of the electromagnetic spectrum, such as thermal damage. Most dramatically, patients (approximately 60–75%) [1] can experience neuronal blockade immediately upon application and have a resulting dramatic pain reduction even if they have had neuropathic pain symptoms for years. Interestingly, it is thought that the neuronal blockade effect may potentiate the peripheral reconditioning of the CNS in terms of long-term pain control.

The FDA clearance of the first low-frequency pulsed magnetic field device (mPNS) for treating pain associated with diabetic neuropathy is a significant milestone in the clinical acceptance of this treatment modality [2]. The device was found to be both safe and effective in a group of patients who had historically had little success with other pain control methods. Painful diabetic neuropathy (PDN) is one of the most common complications of diabetes mellitus, affecting 50% of patients with the disease, and represents a significant morbidity [3]. However, effective treatments for pain relief in diabetic neuropathy have been minimal. This clearance may therefore have far-reaching implications for a wide range of chronic pain conditions.

In this review, we examine our current understanding of the mechanisms of action of low-frequency pulsed magnetic fields in the treatment of pain. We will review the evidence for the various mechanisms proposed by preclinical studies, clinical trials, and theoretical models. By examining these different mechanisms, we can both learn about how this therapy may be most efficacious and begin to understand its potential use cases for treating these conditions, as well as others.

### 1.1. Characteristics and Comparative Benefits of LFPMFs

#### 1.1.1. Frequency and Field Properties

As the name implies, low-frequency pulsed magnetic fields are characterized by the frequency at which the fields are applied. Typically, this frequency range is considered to be from 0.5 Hz to 100 Hz, although some sources use 300 Hz as the upper limit of the range. To qualify as a pulsed field, the electromagnetic field is not constant but rather is applied in pulses. The specific properties of the pulses (width, rise time, duty cycle) can be modified depending on the intended treatment.

In addition to the frequency of the field, the field strength is also essential when considering the physical characteristics of LFPMFs. The traditional field strength of LFPMFs used in wound healing is typically very low, ranging from 1 to 100 milliTesla (mT). This is far below the levels that can even cause thermal effects, much less tissue damage. LFPMFs for pain control have only been substantiated in the literature with RCTs with stronger magnetic fields ranging up to 1.6T [1,2,4,5,6] using mPNS devices. Only stronger, more focused magnetic fields can generate action potentials in nerves. The low frequency also allows the magnetic field to penetrate more deeply into the tissues than would be possible with other forms of electromagnetic energy, which are absorbed more readily by the superficial tissues.

#### 1.1.2. Benefits of Magnetic Stimulation Modalities

Polson was the first to report magnetic stimulation of peripheral nerves in 1982 [7]. In his experiments, Polson demonstrated the painless nature of magnetic stimulation as well as the ability of these fields to stimulate deep nerves. This was the first step in developing an understanding of the significant benefits that magnetic field therapy has over other electrical stimulation modalities, such as transcutaneous electrical nerve stimulation (TENS) and other forms of electrical therapy. The significant difference between magnetic and electric field therapies lies in the fundamental distinction between a magnetic field and an electric field, as well as their interaction with biological tissues.

A magnetic field can penetrate tissues far more deeply than is possible with electric fields. The electric current can be applied to the skin surface but must then pass through the high-resistance stratum corneum, and is heavily attenuated by the underlying tissues. In contrast, the magnetic field can pass deeply into the body with minimal attenuation. This allows the LFPMFs to affect deeper neural structures and tissues, which could not be treated by surface electrical stimulation.

The magnetic field provides a broader and more homogeneous field distribution than the electric field. The electric current will take the path of least resistance through tissues, resulting in a non-uniform field distribution with areas of high and low current density. The magnetic field, on the other hand, passes through tissues relatively uniformly, regardless of the type of tissue or its resistance. This results in more consistent treatment of the target tissues and minimizes the possibility of areas of either over-stimulation or under-stimulation. In addition, LFPMF therapy is entirely non-invasive. It does not require contact with the skin, eliminating concerns about skin irritation, proper electrode placement, and patient discomfort, as is the case with electrical stimulation. This makes LFPMF therapy particularly attractive in patients where electrode placement would be difficult or contraindicated, such as those with wounds or burns.

## 2. Mechanisms of Action in Pain Control

### 2.1. Neuronal Blockade and Sensory Inhibition

One of the most striking and clinically significant mechanisms of LFPMF action in pain control is the induction of neuronal blockade, which produces rapid sensory inhibition. Clinical observations have documented that approximately 60–75% of patients experience immediate pain relief following LFPMF treatment, even in cases of long-standing neuropathic pain that has persisted for decades [1]. This rapid onset of effect suggests a direct action on neural transmission rather than a process requiring cellular or molecular changes that would take longer to manifest.

The mechanism of this neuronal blockade involves the induction of electrical currents in neural tissues through electromagnetic induction. According to Faraday’s law, a time-varying magnetic field induces an electric field in conductive materials, including biological tissues. When these induced electric fields interact with neuronal membranes, they can alter the membrane potential and influence the generation and propagation of action potentials.

One perceived initiation of chronic neuropathic pain is the loss of inhibitory function in Aβ nerve fibers, often due to the degradation or damage of the myelin sheath. Magnetic fields pass through soft tissue unobstructed and, therefore, can be targeted directly at the Aβ nerves. Delivering stimulation at 0.2–5 Hz with a precise pulse-width (240–290 μs) specifically activates Aβ nerve fibers. A signal with a frequency and pulse-width outside of this range cannot recruit Aβ fibers [4]. The equipment used consists of a high-current pulse generator capable of producing a large electric discharge current (several thousand amperes). The current flows through a stimulating coil, generating magnetic pulses with a field strength of up to several Tesla. Heat is an unavoidable by-product derived from magnetic pulse generation; therefore, the coil must be contained in a liquid-cooled system. Many types of coils have been manufactured. Two frequently used coils are the round coil and the figure-eight coil. Axon Therapy uses a figure-of-eight coil that produces a stronger magnetic field at its center, with precise focus. This device is an mPNS device.

### 2.2. Effects on Voltage-Gated Ion Channels

At the cellular level, the induced electric fields can affect voltage-gated ion channels that are critical for action potential generation and propagation [5,8,9,10,11]. LFPMFs have been shown to modulate the activity of various ion channels, including voltage-gated sodium, potassium, and calcium channels. The modulation of sodium channel activity can reduce neuronal excitability by decreasing the availability of channels for activation or by altering their kinetics.

Studies have demonstrated that exposure to pulsed magnetic fields can alter the voltage dependence of channel activation and inactivation, effectively increasing the threshold for action potential generation [6]. This effect would be particularly relevant for nociceptive neurons, which transmit pain signals from the periphery to the central nervous system. By reducing the excitability of these neurons, LFPMFs can effectively block or attenuate the transmission of pain signals.

### 2.3. Membrane Potential Hyperpolarization

Another mechanism contributing to neuronal blockade involves the hyperpolarization of neuronal membranes [12,13,14]. LFPMFs can influence the resting membrane potential, reducing the likelihood that neurons will reach threshold for generating an action potential. The hyperpolarization effect may result from modulation of potassium channel activity, particularly “leak channels” that help establish the resting membrane potential. By increasing potassium efflux or decreasing sodium influx, LFPMFs can shift the membrane potential in a more negative direction, effectively reducing neuronal excitability.

### 2.4. Central Nervous System Reconditioning

Beyond the immediate effects of neuronal blockade, LFPMFs appear to promote longer-term changes in pain processing through what has been termed peripheral reconditioning of the central nervous system [15]. This concept acknowledges that chronic pain encompasses not only peripheral sensitization of nociceptors but also central sensitization, where changes in the central nervous system can amplify and prolong pain signals even after the initial injury has healed. It is well recognized that when pain persists, the nervous system undergoes structural and functional changes, and this central sensitization can lead to “wind-up”.

The initial neuronal blockade produced by LFPMFs provides a window of reduced pain signaling to the central nervous system [15]. During this period, the absence of or reduction in nociceptive input allows central pain processing circuits to begin normalizing their function. This process involves reversing maladaptive neuroplastic changes that occur with chronic pain, including changes in synaptic strength, altered expression of neurotransmitter receptors, and modifications in descending pain modulation systems.

### 2.5. Modulation of Synaptic Plasticity

Chronic pain is associated with long-term potentiation of pain pathways in the spinal cord and brain. This process increases the efficiency of synaptic transmission and contributes to pain amplification. Evidence suggests that LFPMFs may help reverse these changes by promoting long-term depression of pain-related synapses. This effect may involve modulation of glutamate receptor activity, particularly NMDA receptors, which play a crucial role in synaptic plasticity.

The electromagnetic fields may also influence the release and reuptake of neurotransmitters at pain-processing synapses. By normalizing neurotransmitter dynamics, LFPMFs could help restore standard pain processing and reduce the amplification of pain signals that characterizes central sensitization. This mechanism would explain the sustained pain relief observed in many patients following a course of LFPMF treatment, even after the treatment has been discontinued [9].

### 2.6. Enhancement of Descending Inhibition

The central nervous system possesses endogenous pain modulation systems that can inhibit pain signal transmission at the spinal cord level. These descending inhibitory pathways originate in brain regions such as the periaqueductal gray and rostral ventromedial medulla and release neurotransmitters, including serotonin, norepinephrine, and endogenous opioids, to reduce pain transmission [16]. Dysfunction of these inhibitory systems contributes to chronic pain states.

Evidence suggests that LFPMFs may enhance the function of descending inhibitory pathways [11]. This enhancement could occur through several mechanisms, including increased release of inhibitory neurotransmitters, upregulation of their receptors in the spinal cord, or increased activity of inhibitory neurons in the brain. By strengthening endogenous pain inhibition, LFPMFs would provide sustained pain relief that persists beyond the immediate treatment period.

### 2.7. Effects on Neuropathic Pain

Neuropathic pain, resulting from damage or dysfunction of the central and/or peripheral nervous system, represents one of the most challenging pain conditions to treat. The effectiveness of LFPMFs in neuropathic pain, as demonstrated in diabetic neuropathy and other neuropathic conditions, warrants particular attention to the mechanisms specific to this type of pain.

Neuropathic pain involves several pathophysiological mechanisms, including ectopic firing of damaged nerves, altered expression and distribution of ion channels, inflammatory changes in nerve tissues, and central sensitization. LFPMFs may address multiple aspects of this complex pathophysiology. The ability to reduce ectopic firing by modulating ion channel activity is particularly relevant, as spontaneous firing from damaged nerves is a major contributor to neuropathic pain [4].

## 3. Clinical Evidence and Applications

### 3.1. Pain Management Applications

Clinical studies of LFPMFs (mPNS) for pain management have consistently demonstrated significant therapeutic efficacy across various pain conditions [1,2,4,17,18]. The FDA approval for diabetic neuropathy pain was based on rigorous clinical trials that established both a significant reduction in pain intensity and excellent tolerability with no reported serious adverse effects. Patients in these trials typically experienced a significant reduction in pain within just a few treatment sessions, and these pain-relief benefits were maintained over extended follow-up periods.

The clinical response rate of 60–75% for immediate pain relief after treatment is a highly robust finding, given the high probability of treatment failure in populations that have failed multiple previous treatment attempts. Moreover, long-term follow-up in published trials has shown that pain reduction is durable and can be maintained for months after treatment completion, supporting the hypothesis of neuroplastic changes and CNS reconditioning beyond mere temporary symptomatic improvement.

The potential benefits of LFPMF therapy for other chronic pain conditions beyond diabetic neuropathy have been suggested by preliminary clinical evidence.

The initial studies have been well designed, even with a sham study, and are all statistically significant. They support continued clinical investigation of LFPMF therapy for chronic pain more broadly, given the favorable safety profile and strong preliminary evidence in diabetic neuropathy.

A narrative review discussed the high incidence of chronic pain syndromes in the post and the long COVID period [19]. It was thought that post-COVID chronic pain might include a newly developed chronic pain as a part of post-viral syndrome; worsening of preexisting chronic pain due to the associated changes in the medical services. One Case report has been reported on pulsed electromagnetic field therapy in a long COVID patient and speculated that individualized PEMF may resolve post-infectious symptoms [20].

### 3.2. Treatment Parameters and Optimization

The efficacy of LFPMF therapy depends on several treatment parameters, including field frequency and intensity, pulse characteristics, treatment duration, and treatment frequency. Optimization of these parameters is necessary to maximize therapeutic effects, ensure patient safety, and maintain treatment practicality.

### 3.3. Treatment Duration and Frequency

Protocols for treatment duration and frequency vary considerably across different studies and applications. This variation, combined with the potential for individual patient variability in response, makes it difficult to define the optimal treatment duration and frequency for specific applications. The treatment protocols for modern studies that differentiate magnetic peripheral nerve stimulation (mPNS) from other electrical (ePNS) or transcranial stimulation (rTMS) have been standardized. These protocols specify three treatments in a row daily, followed by three weekly treatments, equaling six treatments in the first month. The study protocols then proceed to a bi-weekly therapy in month two and thereafter a monthly maintenance schedule. Results were reproducible in multiple sites in the country with multiple providers. The dosing was 0.5 Hz in all cases with a fixed pulse width of 290 ms, delivering 400 pulses for the 13.33 min therapy. This protocol was developed by Leung [4] after testing for the refractoriness period of the nerves. Study patients for the initial FDA clearance paper all had neuromas. The VA observational real-world study included patients with post-surgical neuropathy, post-traumatic neuropathy, post amputee stump pain, phantom limb pain, post thoracotomy pain, painful diabetic neuropathy, idiopathic neuropathy and greater occipital neuralgia. The two multisite, crossover RCTs followed the FDA indications of chronic intractable neuropathic pain, post-traumatic, post-operative, and included diabetic neuropathic pain patients. Only the most recently published 2025 paper on PDN dealt with a homogenous group of patients.

Patients with radicular pain due to disk herniation are being treated with a location determined by mapping, corresponding to the affected dorsal root ganglion. They receive 4–6 treatment sessions spanning a weekly schedule.

Low-energy devices usually require treatment sessions of 20–60 min, typically lasting 2–4 weeks. Some devices, such as the Scrambler^TM^, require 10 consecutive treatments. Some patients experience significant pain relief with a shorter course of treatment, while others require longer or repeated courses to maintain benefits.

Treatment protocols for wound healing applications typically also involve daily treatment sessions of 30–60 min. Protocols may vary in whether they continue treatment until wound closure or for a fixed period. Some protocols use twice-daily therapies for more severe or slowly healing wounds. The optimal treatment duration and frequency likely vary depending on wound characteristics, patient factors, and the specific healing deficit being targeted.

### 3.4. Safety Considerations

LFPMF therapy has an excellent safety record with and a significant clinical advantage over many pharmacological treatments. Most medications have associated troublesome side effects and/or contraindications, but LFPMFs rarely cause any problems. The electromagnetic fields are non-ionizing; therefore, no charge buildup occurs under the skin, and their low frequencies and field strengths do not cause any thermal damage or tissue injury.

Clinical trials and the extensive clinical experience to date have documented few adverse events or side effects associated with LFPMF treatment. The most commonly reported side effects are mild and transient in nature. Patients sometimes experience temporary tingling or warmth at the treatment site. These effects typically resolve quickly and usually do not require cessation of the treatment. No serious adverse events have been consistently linked to LFPMF therapy in well-conducted clinical trials. The earlier transcranial (TMS) studies showed excellent safety parameters as well [19,20].

Only a few contraindications to LFPMF therapy have been identified. The most significant is in patients with implantable cardioverter-defibrillators, or other active implanted medical devices. These patients are generally precluded from LFPMF treatment due to the theoretical risk of device interference. Pregnant women are typically also excluded as a precautionary measure. The devices are not placed over the head or the heart. There is no evidence of harm, but adequate data do not exist for this population.

### 3.5. Future Directions and Research Needs

While significant progress has been made in understanding the mechanisms and potential clinical applications of LFPMFs, several important research questions remain to be addressed in future studies. A more thorough understanding of the cellular and molecular mechanisms underlying LFPMF effects will be required to rationally optimize treatment parameters and identify patient populations most likely to benefit from this therapy.

Future research will focus on RCTs evaluating the use of mPNS for osteoarthritic and perioperative applications. Posters have been presented at the North American Neuromodulation Society (NANS) annual meeting 2024. A pilot study publication is in submission. The use of mPNS to stimulate S3 for overactive bladder has also been studied in a pilot study, which is being prepared for publication. This would enable a non-interventional, non-surgical technique to provide the same excellent response rates as the implanted alternative. A cost-effectiveness paper is also in publication showing a significant decrease in medical expenditure on a nonimplanted device such as mPNS. Future Biomaterials engineering will look at different sized coils for different applications such as head and neck.

The potential for combination therapies is also particularly intriguing. LFPMFs could be combined with a wide variety of other treatments, including pharmacological treatments, cell-based therapies, growth factor administration, regenerative-type injections, or physical rehabilitation, to achieve synergistic effects. Research into such combinations could lead to the development of novel treatment paradigms that provide superior outcomes to any single therapy alone.

## 4. Conclusions

Low-frequency pulsed magnetic fields offer a promising non-invasive therapeutic modality for managing chronic neuropathic pain. The therapeutic effects of LFPMFs can be rationalized by their impact on multiple complementary pathways, simultaneously modulating neural activity to achieve pain relief while also enhancing cellular processes that drive tissue repair. The immediate neuronal blockade provides fast pain relief, while the downstream CNS reconditioning helps maintain these effects in the long term. Practically, magnetic fields also have distinct advantages in that they penetrate more deeply into tissue and are more evenly distributed than electrical currents. Paired with an outstanding safety record and minimal contraindications, LFPMF therapy is often accessible to many more patients for whom conventional treatments are not possible.

Clinical evidence for the efficacy of LFPMFs is growing, particularly for neuropathic pain, with FDA clearance for diabetic neuropathy being a significant regulatory milestone. Strong preclinical evidence appears to support wound healing applications [21,22,23,24,25,26]; however, more extensive clinical trials are needed to establish efficacy in various types of wounds definitively. The alignment of mechanistic understanding, preclinical evidence, and clinical results makes a compelling case for broader adoption of LFPMF therapy.

Future research should focus on optimizing treatment parameters for specific indications, identifying patient populations most likely to benefit, such as chemotherapy-induced neuropathic pain, overactive bladder, and fecal incontinence. The longest mPNS follow-up study is at one year. Expanded duration follow-up and additional studies are needed to confirm if mPNS can provide a permanent “reset” of the pain processing system. As our understanding of LFPMFs continues to evolve, this technology has the potential to become a crucial component of the therapeutic armamentarium for pain and wound management, providing a safe and effective option for patients suffering from these challenging conditions.

## Data Availability

No new data were created or analyzed in this study.

## Abbreviations

The following abbreviations are used in this manuscript:

LFPMF	Low Frequency Pulsed Magnetic Field
FDA	Food and Drug Administration
mPNS	Magnetic Peripheral Nerve Stimulation
PDN	Painful Diabetic Neuropathy
TENS	Transcutaneous Electrical Nerve Stimulation
